# Endothelial cells induce cancer stem cell features in differentiated glioblastoma cells via bFGF

**DOI:** 10.1186/s12943-015-0420-3

**Published:** 2015-08-19

**Authors:** Evelyn Fessler, Tijana Borovski, Jan Paul Medema

**Affiliations:** Laboratory for Experimental Oncology and Radiobiology (LEXOR), Center for Experimental Molecular Medicine (CEMM), Academic Medical Center (AMC), University of Amsterdam, Amsterdam, Meibergdreef 9, 1105 AZ Amsterdam, The Netherlands; Present address: Department of Surgery, University Medical Center (UMC) Utrecht, Utrecht, The Netherlands

**Keywords:** Glioblastoma multiforme, Cancer stem cells, Tumor microenvironment, Dedifferentiation

## Abstract

**Background:**

Glioblastoma multiforme (GBM) is a rapidly growing malignant brain tumor, which has been reported to be organized in a hierarchical fashion with cancer stem cells (CSCs) at the apex. Recent studies demonstrate that this hierarchy does not follow a one-way route but can be reverted with more differentiated cells giving rise to cells possessing CSC features. We investigated the role of tumor microvascular endothelial cells (tMVECs) in reverting differentiated glioblastoma cells to CSC-like cells.

**Methods:**

We made use of primary GBM lines and tMVECs. To ensure differentiation, CSC-enriched cultures were forced into differentiation using several stimuli and cultures consisting solely of differentiated cells were obtained by sorting on the oligodendrocyte marker O4. Reversion to the CSC state was assessed phenotypically by CSC marker expression and functionally by evaluating clonogenic and multilineage differentiation potential.

**Results:**

Conditioned medium of tMVECs was able to replenish the CSC pool by phenotypically and functionally reverting differentiated GBM cells to the CSC state. Basic fibroblast growth factor (bFGF), secreted by tMVECs, recapitulated the effects of the conditioned medium in inducing re-expression of CSC markers and increasing neurosphere formation ability of differentiated GBM cells.

**Conclusions:**

Our findings demonstrate that the CSC-based hierarchy displays a high level of plasticity showing that differentiated GBM cells can acquire CSC features when placed in the right environment. These results point to the need to intersect the elaborate network of tMVECs and GBM CSCs for efficient elimination of GBM CSCs.

**Electronic supplementary material:**

The online version of this article (doi:10.1186/s12943-015-0420-3) contains supplementary material, which is available to authorized users.

## Background

Glioblastoma multiforme (GBM) is a nearly universally lethal, heterogeneous primary brain tumor. Despite aggressive treatment comprising surgical resection, radiotherapy, and temozolomide (TMZ) chemotherapy, the prognosis of GBM remains dismal with a 5-year survival rate of only 10 % [1]. After treatment, recurrence is almost inevitable and is thought to be driven by cancer stem cells (CSCs). CSCs are suggested to be the only cells within a malignancy able to drive tumor growth and progression. Moreover, they have been shown to resist most cancer therapies and are therefore held responsible for poor clinical outcome [2–4].

The CSC hypothesis suggests that tumors follow a hierarchical organization with CSCs localizing at the apex and giving rise to all differentiated progeny that can be found in the malignancy. However, several studies have challenged this concept indicating that the differentiation of CSCs is not a one-way route but might be a reversible process that can be directed by signals from the tumor microenvironment [5–7]. It should be noted that the dynamic conversion between differentiation states could be, at least in part, an intrinsic feature. A model of this stochastic interconversion was reported by Gupta and colleagues for breast cancer [8] and was also shown in melanoma [9]. As for GBM, CSCs can be generated by progenitor cells in response to microenvironmental signals [10–12]. Two recent studies showed that non-stem glioblastoma cells can convert to CSC-like cells upon therapeutic stress, such as TMZ treatment or ionizing radiation [13, 14]. These results demonstrate that stochastic transition between distinct differentiation states - as has been described before for breast cancer - also occurs in GBM [8, 13, 15, 16]. Differentiated cells were defined by the downregulation of CSC markers and the increased expression of differentiation markers; however, the selection of these cells was not based on proteins flagging the differentiation state. In case of a cell sorting-based approach, differentiated cells were defined by the absence of CSC markers. This approach does not exclude the existence of concealed CSCs not expressing the classical CSC markers which could expand upon treatment.

In this study, we assessed the role of the GBM CSC niche consisting of tumor microvascular endothelial cells (tMVECs) in regulating cell fate. Conditioned medium of tMVECs was sufficient to phenotypically and functionally revert differentiated cancer cells to the CSC state. To avoid the presence of cryptic CSCs, we forced primary GBM spheroid cultured cells to differentiate to distinct lineages by multiple differentiation techniques. Additionally, with the use of the cell surface marker O4, we sorted differentiated oligodendrocytes from the total population and therefore excluded the presence of CSCs devoid of traditional CSC markers. O4-expressing differentiated GBM cells were able to convert to CSC-like cells, showing that differentiated cancer cells could acquire CSC features.

## Results

### tMVECs increase self-renewal capacity and CD133 expression of CD133^−^ GBM cells

**Fig. 1:**
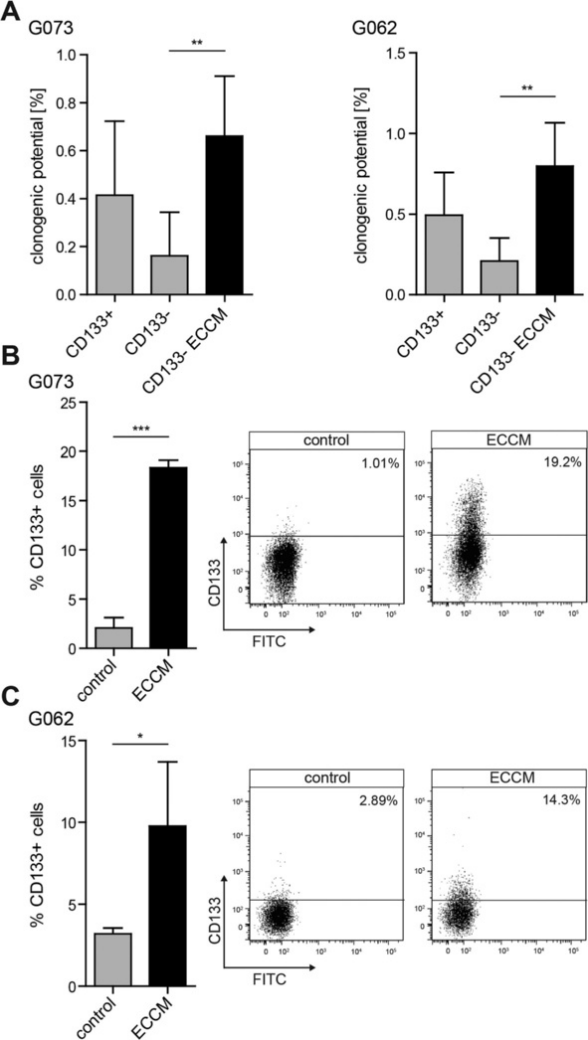
ECCM increases the clonogenic potential and CD133 expression of CD133^−^ GBM cells. **a** Indicated is the clonogenic potential of the CD133^−^ fraction of G073 (left) and G062 (right) cultures in control medium or ECCM and of CD133^+^ cells in control medium, scored 2 weeks after sorting (*n* ≥ 3). **b**, **c** G073 **(b)** and G062 **(c)** CD133^−^ cells were plated in control medium or ECCM directly after sorting. Quantification and FACS profiles depict CD133 expression 24 h (G073) or 72 h (G062) after sorting (*n* = 3)

Populations expressing high levels of CD133 were shown to be enriched for CSCs in a wide variety of malignancies, including GBM [17, 18]. Although it is questionable whether CD133 identifies the CSC population in all GBM tumors, [19–22] its usefulness to identify cells that possess the capacity to self-renew, to spawn all differentiated progeny, and to serially propagate tumors has been well-documented in several primary spheroid cultured GBM lines [17, 18]. We therefore examined the expression of CD133 in three primary GBM lines, G073, G062, and G408. As described before, such GBM spheroid cultures displayed a gradient of CD133 expression and the highest CD133 expressing cells (top 10 %, CD133+) were endowed with high self-renewal capability determined by clonogenic assays [17, 23]. In contrast, the clonogenicity of cells expressing no or low levels of CD133 (lowest 10 %, CD133-) was lower, indicating their more differentiated character (Fig. 1a and Additional file 1: Figure S2B). Although these CD133^−^ cells had a lower capacity to self-renew and be clonogenic, conditioned medium derived from tMVECs (endothelial cell conditioned medium, ECCM) was capable of conveying such CSC features to these non-stem GBM cells. When plated in ECCM, the self-renewal ability of CD133^−^ cells increased to a similar clonogenic potential as the one of CD133^+^ cells (Fig. 1a and Additional file 1: Figure S2B), suggesting that factors present in ECCM induce self-renewal in initially poorly clonogenic cells.

To address the question whether this effect is also observed when employing other CSC markers, we made use of the G073 spheroid culture, which expresses a gradient of stage-specific embryonic antigen 1 (SSEA-1), a cell surface marker that has been reported to identify GBM CSCs [24]. The low clonogenic potential of SSEA-1^−^ cells in control medium strongly increased when these cells were plated in ECCM (Additional file 1: Figure S2A), indicating that the ECCM effect is not specific for CD133.

We speculated that the 2 week time frame of clonogenic assays might leave possible small numbers of contaminating CD133^+^ cells in the sorted CD133^−^ fraction enough time to proliferate and grow out, potentially misjudging the effect of ECCM as reversion of CD133^−^ cells instead of outgrowth of CD133^+^ cells. To address this point, CD133^−^ cells were sorted in control medium or ECCM and CD133 expression was analyzed 24 or 72 h after sorting, giving no time for possible contaminating CD133^+^ cells to grow out. ECCM was able to increase expression of CD133 in the initially negative population already 24 or 72 h after plating (Fig. 1b and c and Additional file 1: Figure S2C).

The above-described effects could also be observed when CD133^−^ G062 cells were plated in conditioned medium derived from human umbilical vein endothelial cells (HUVECs; HUVEC conditioned medium, HCM) (Additional file 1: Figure S2D and S2E).

Thus, tumor-derived as well as normal tissue endothelial cells are capable of phenotypically and functionally reverting non-stem GBM cells to the CSC state.

### tMVECs induce CD133 expression in differentiated GBM cells

**Fig. 2:**
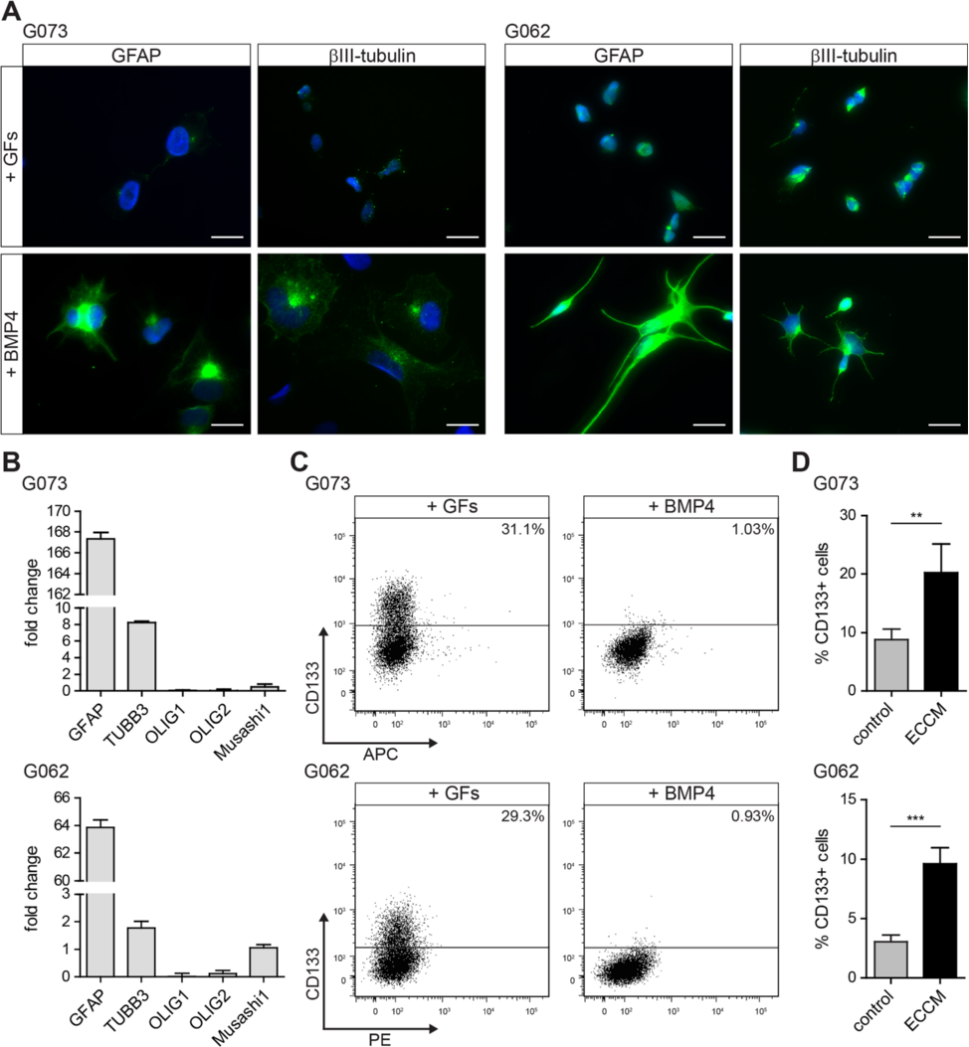
Differentiation of GBM CSCs using BMP4 leads to upregulation of differentiation markers and downregulation of the CSC marker CD133 which is reversed by ECCM. **a** BMP4 induces upregulation of the astrocyte marker GFAP in G073 (left) and G062 (right) cells and induction of the neuronal marker βIII-tubulin in G073 cells as compared to cells plated in CSC medium + GFs (scale bars 20 μm). **b**-**d** Upper panels: G073, lower panels: G062. **b** Differentiation markers are upregulated and CSC markers are downregulated upon BMP4 treatment compared to cells plated in CSC medium + GFs as determined by qRT PCR (1 representative of 3 independent experiments is shown) and **c** the CSC marker CD133 is not detectable anymore (*n* > 3). **d** BMP4-differentiated CD133^−^ cells were sorted and plated in either control medium or ECCM. 5 days after sorting CD133 expression was reanalyzed by FACS (*n* ≥ 4)

Although CD133^−^ cells are considered to comprise the more differentiated part of a GBM spheroid culture, they are not differentiated and can thus contain a subpopulation of cells that still maintain self-renewal and sphere forming activity. Therefore, in order to determine the effect of ECCM on differentiated tumor cells, we forced *in vitro* differentiation of GBM CSCs toward the neuronal and astrocytic lineages using bone morphogenetic protein 4 (BMP4) [25]. After 7 days of BMP4 treatment, the G073 and G062 primary GBM lines displayed glial fibrillary acidic protein (GFAP) expression. G073 cells also induced βIII-tubulin expression and downregulated the CSC marker SSEA-1 (Fig. 2a and Additional file 1: Figure S3A). Quantitative real-time PCR (qRT PCR) results confirmed the increased expression of these differentiation markers and revealed the downregulation of the CSC marker OLIG2 in both cultures and of Musashi1 in G073 cells (Fig. 2b). In addition, CD133 and Nestin expression were strongly reduced on BMP4-treated GBM cells (Fig. 2c and Additional file 1: Figure S3B).

Even though GBM cells were almost completely devoid of CD133 upon BMP4 treatment, we sorted CD133^−^ cells to avoid the presence of cells expressing low levels of this CSC marker. Differentiated, CD133^−^ cancer cells were sorted into control medium or ECCM and CD133 expression was reanalyzed after 5 days revealing that cells plated in ECCM significantly induced CD133 expression (Fig. 2d). BMP4-differentiated CD133^−^ G073 and G062 cells were plated in clonogenic assays to address the functional conversion of these differentiated cells to the CSC state. Indeed, the clonogenic potential of these cells increased when plated in ECCM compared to control medium (Additional file 1: Figure S3C).

Hence, based on marker expression and on clonogenic capacity, ECCM is capable of reverting BMP4-differentiated cells to the CSC state.

### bFGF secreted by tMVECs induces the reversion of differentiated GBM cells

**Fig. 3:**
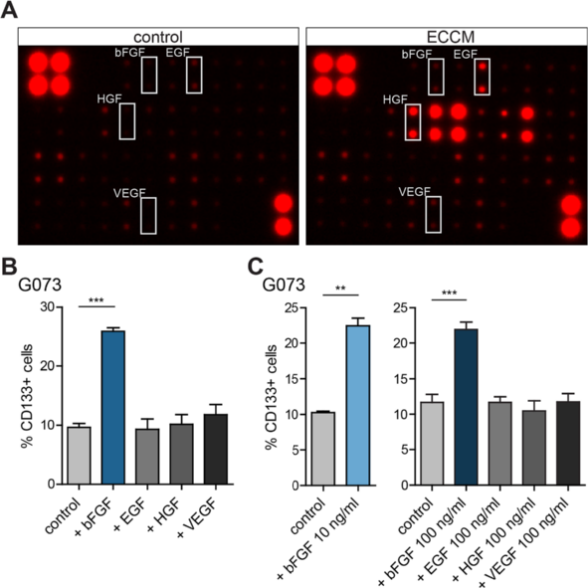
bFGF in ECCM induces CD133 expression on BMP4-differentiated CD133^−^ cells. **a** Growth factor array comparing control medium (left panel) to ECCM (right panel). **b**, **c** Differentiation of G073 cells using 100 ng/ml BMP4 for 7 days and subsequent sorting of CD133^−^ cells. Graphs show % CD133^+^ cells 5 days after sorting, as determined by FACS ((**b**) *n* = 3, (**c**) left *n* = 2, right *n* = 3)

We performed a growth factor array on ECCM to identify which factors secreted by tMVECs could be responsible for the reversion. This revealed a wide range of growth factors (GFs) that could potentially be involved in reversal of differentiated GBM cells to the CSC state (Fig. 3a and Additional file 1: Figure S4A). We focused on bFGF, epidermal growth factor (EGF), hepatocyte growth factor (HGF), and vascular endothelial growth factor (VEGF) in further experiments, since bFGF and EGF play an important role in GBM CSC maintenance, [26, 27] HGF was shown to revert differentiated colorectal cancer cells to a CSC phenotype, [5] and VEGF was described to promote viability of GBM CSCs [28].

To assess which factor could be responsible for induction of CD133 expression, BMP4-differentiated CD133^−^ G073 cells were sorted and plated in control medium, medium containing bFGF, EGF, HGF, or VEGF. Analysis of CD133 expression 5 days after plating unveiled that only bFGF significantly increased the amount of CD133-expressing cells (Fig. 3b). Moreover, when G062 cells were induced to differentiate with BMP4, stimulation of the resulting CD133^−^ cells with bFGF also significantly re-induced CD133 expression, indicating that the reversion was observed in other primary cultures as well (Additional file 1: Figure S4B). The observed effect of bFGF on induction of CD133 expression was not concentration dependent, as 10, 50, and 100 ng/ml yielded comparable results (Fig. 3c and Additional file 1: Figure S4B). Additionally, EGF, HGF, and VEGF were not able to induce CD133 expression even when used at very high doses (100 ng/ml, Fig. 3c).

**Fig. 4:**
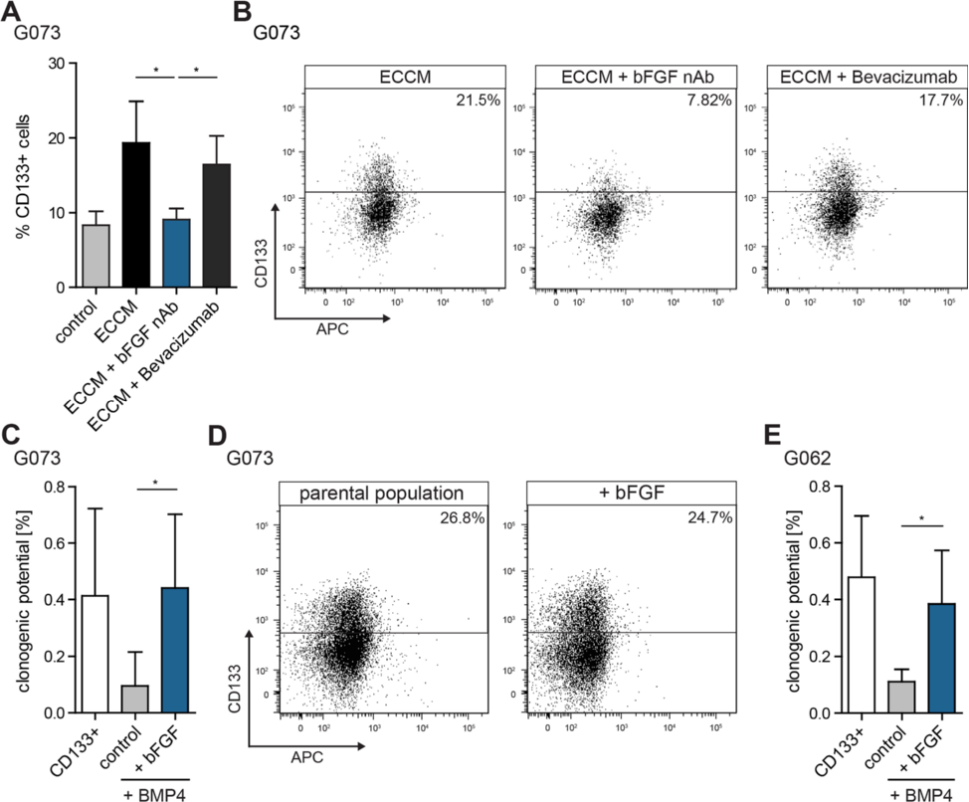
bFGF in ECCM is responsible for induction of CD133 expression and increases the clonogenic potential of BMP4-differentiated CD133^−^ cells. **a** BMP4-differentiated CD133^−^ G073 cells were sorted and plated in control medium, ECCM, ECCM containing a bFGF neutralizing antibody (bFGF nAb), or ECCM containing Bevacizumab. 5 days after sorting CD133 expression was reanalyzed by FACS (*n* = 3). **b** FACS plots corresponding to (**a**). **c** The clonogenic potential of BMP4-differentiated CD133^−^ G073 cells was assessed using clonogenic assays (*n* = 6). **d** CD133 expression of spheres formed in (**c**) in the bFGF condition compared to the non-differentiated parental G073 spheroid culture (*n* = 2). **e** The clonogenic potential of BMP4-differentiated CD133^−^ G062 cells was assessed using limiting dilution assays (*n* = 5)

To prove that bFGF present in the conditioned medium was responsible for the ECCM-induced reversion we made use of a bFGF neutralizing antibody (bFGF nAb). Addition of this antibody to ECCM prevented the induction of CD133 expression in BMP4-differentiated G073 cells (Fig. 4a and b). In contrast, the addition of Bevacizumab, which targets VEGF, did not affect the induction of CD133 expression (Fig. 4a and b).

The growth factor array performed revealed additional interesting candidates, such as insulin-like growth factor-binding proteins (IGFBPs) and specifically IGFBP-2, which has previously been implicated in GBM CSC biology [29]. Our results demonstrate that bFGF is capable of reverting differentiated GBM cells to the CSC state in the experimental model used. It should be noted, however, that under different circumstances other proteins might fulfil similar functions.

To determine whether bFGF also functionally reverts differentiated GBM cells to the CSC state, BMP4-differentiated CD133^−^ cells were sorted in clonogenic assays, either in control or bFGF-containing medium. Indeed, bFGF was capable of promoting self-renewal capacity yielding a clonogenic potential similar to that of CD133^+^ GBM CSCs (Fig. 4c and e). G073 colonies that grew out in medium containing bFGF entirely resembled the parental undifferentiated population as judged by CD133 expression (Fig. 4d).

Moreover, the expression of the differentiation markers GFAP and βIII-tubulin was downregulated to the level of the undifferentiated parental population in G062 cells collected from the bFGF condition of the clonogenic assay (Additional file 1: Figure S3D). Additionally, cultures derived from BMP4-differentiated CD133^−^ G062 cells plated in bFGF containing medium could recapitulate the same differentiation pattern as the parental population, indicating their reversion to the CSC state (Additional file 1: Figure S5).

These results demonstrate that bFGF in ECCM is able to phenotypically and functionally revert differentiated GBM cells to CSC-like cells.

### bFGF induces the reversion of O4^+^ oligodendrocyte-like cells to the CSC state

**Fig. 5:**
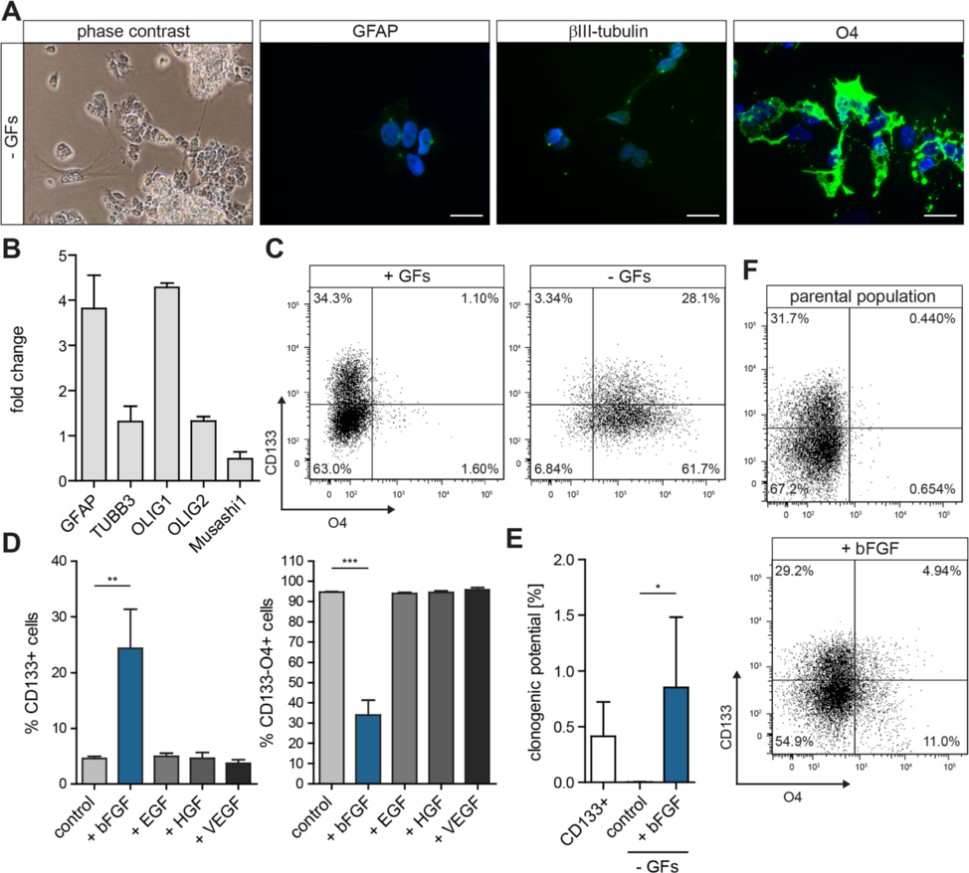
Differentiated GBM oligodendrocytes can be phenotypically and functionally redirected to the CSC state by bFGF. **a** Differentiation of G073 cells for 7 days by GF withdrawal (− GFs). Differentiation induces morphological changes and strong upregulation of the oligodendrocyte marker O4 (scale bars 20 μm). **b** Analysis of differentiation and CSC markers by qRT PCR. Depicted is the fold change compared to cells plated in CSC medium + GFs. 1 representative of 3 independent experiments is shown. **c** Differentiated GBM cells express the oligodendrocyte marker O4 on their surface as determined by FACS. **d** CD133^−^O4^+^ cells were sorted and plated in the indicated condition. 5 days after sorting CD133 and O4 expression were reanalyzed by FACS (*n* = 3). **e** The clonogenic potential of differentiated CD133^−^O4^+^ cells was determined using clonogenic assays (*n* = 4). **f** CD133 and O4 expression of spheres formed in (**e**) in the bFGF condition compared to the non-differentiated parental GBM spheroid culture (*n* = 3)

The identification of differentiated cells after BMP4 treatment relies on the absence of the CSC marker CD133. However, tumorigenic potential has also been described for CD133^−^ glioblastoma cells and various other markers besides CD133 could identify populations possessing CSC features as well [19–22, 24, 30]. Furthermore, the selection of differentiated cells by the absence of a CSC marker does not exclude the presence of concealed CSCs. In a population lacking classical CSC markers these cryptic CSCs could be activated and reconstitute the distinct cell fractions present in the original population. To assess the reversion potential of a culture consisting exclusively of differentiated cells we made use of the cell surface oligodendrocyte marker O4. G073 GBM CSCs could be effectively differentiated toward the oligodendrocytic lineage using GF withdrawal (− GFs) or addition of 2 % fetal calf serum (+2 % FCS) for 7 days. In both conditions, cells displayed a differentiated morphology, gained O4 expression and downregulated the expression of Nestin (Fig. 5a, Additional file 1: Figure S6A and Additional file 1: Figure S7). Moreover, cells differentiated with 2 % FCS showed induction of GFAP and βIII-tubulin expression (Additional file 1: Figure S6A). Differentiation into distinct lineages was confirmed by qRT PCR which also revealed the downregulation of the CSC marker Musashi1 in both conditions and of OLIG2 in cells differentiated with 2 % FCS (Fig. 5b and Additional file 1: Figure S6B). As O4 is a cell surface marker detectable by FACS (Fig. 5c and Additional file 1: Figure S6C) the differentiated oligodendrocyte-like tumor cells could be sorted from the total population. Of note is the appearance of a CD133^+^O4^+^ population upon differentiation. Since this population retains high expression of the CSC marker CD133 it was not subject of the current study; however, this population is intriguing and should be characterized in future experiments.

For both differentiation conditions, CD133^−^O4^+^ cells were sorted and plated in control medium, medium containing bFGF, EGF, HGF, or VEGF for 5 days, after which the presence of CD133^−^O4^+^ cells was analyzed. Also under these conditions only bFGF was able to reduce the amount of CD133^−^O4^+^ cells and to reinstall CD133 expression (Fig. 5d and Additional file 1: Figure S6D). Further proof that reversal could be achieved with cells differentiated using GF withdrawal was obtained by stimulating CD133^−^O4^+^ cells that were sorted twice to exclude contamination by missorting (Additional file 1: Figure S8).

The reversal of CD133^−^O4^+^ cells is not only observed phenotypically, but is also a functional conversion as plating in bFGF-containing medium significantly increased the clonogenic potential of these cells (Fig. 5e and Additional file 1: Figure S6E). Analyzing the neurospheres formed in the bFGF condition unveiled that these cells reverted to a CSC-enriched population similar to the parental population according to CD133 and O4 expression (Fig. 5f and Additional file 1: Figure S6F). Moreover, cultures derived from these spheres displayed the same differentiation pattern as the parental population, indicating their reversion to the CSC state as determined by the acquisition of multilineage differentiation potential (Additional file 1: Figure S9).

The same phenotypical and functional reversal of CD133^−^O4^+^ cells induced by bFGF could be observed when stimulating these cells with ECCM (Additional file 1: Figure S10).

Taken together, these results demonstrate that differentiated GBM cells can acquire CSC features and that this process can be orchestrated by tMVECs.

## Discussion

The unidirectional dogma states that CSCs give rise to progenitor cells, which spawn transit amplifying cells that proliferate quickly and differentiate into all progeny found in a tumor. This hypothesis has lately been challenged by data supporting increased dynamics between cell fractions.

Studies that demonstrate reversion of non-stem cells to CSC-like cells depend on the use of frequently debated CSC markers, such as CD133, to define the differentiated population. However, these fractions do not need to be completely devoid of CSCs, as CD133^−^ cells can be tumorigenic as well, spawning tumors when transplanted into mice [13]. This can be explained by contaminating CD133^+^ cells due to missorting or by the existence of CSCs not defined by classical CSC markers. In the case of GBM this could also be dependent on the origin and/or subtype of the GBM tumor tested. To avoid the presence of cryptic CSCs we therefore employed an additional selection step and assessed reversion of GBM cells forced into differentiation and of a culture consisting solely of differentiated cells by sorting on the oligodendrocyte marker O4. Our results demonstrate that differentiated GBM cells can acquire CSC features. Invariably this implies that the cells maintain their CSC capacities, but that these only become evident when the cells are placed in the right microenvironmental conditions. In other words differentiation as well as cancer stemness is in part determined by a dominant microenvironment in these cases.

The analysis of the bFGF/ECCM-induced reversion *in vivo* could not be addressed in this study as the lines used herein did not display tumor growth following subcutaneous injection. Thus, determining the impact of this plasticity on therapy efficacy warrants further investigation.

It is important to note, that distinct primary spheroid cultured GBM lines might differ in their behavior based on origin and subtype affiliation. We described previously that direct contact between tMVECs and two GBM spheroid lines is necessary for induction of proliferation and conditioned medium was not sufficient to induce these effects [31]. Herein, using two different spheroid-cultured GBM lines, conditioned medium was capable to revert differentiated GBM cells to the CSC state, indicating that secreted factors, specifically bFGF, could provide the necessary input. These differences could be explained by our cultures belonging to different subtypes of GBM tumors that might have distinct requirements from their microenvironment due to distinct sets of mutations [32, 33].

## Conclusions

Previous studies have indicated the importance of GBM CSCs in therapy refractoriness and tumor recurrence. Based on these observations major efforts are invested in eradicating the CSC population. Our findings suggest that targeting the CSC fraction might not be sufficient for effective treatment due to its complex cross-talk with the microvasculature. Under the influence of their niche, differentiated tumor cells could potentially acquire CSC features and re-establish the CSC pool to maintain tumor homeostasis. Thus, targeting CSCs through treatment modalities intersecting the effects of the tumor surrounding might be essential for developing effective therapies.

## Methods

### Cell culture, ECCM and HCM preparation, and differentiation of GBM cells

GBM cells and tMVECs were isolated from patient material as described previously [31]. Human tissues were obtained in accordance with the rules of the medical ethical committee of the AMC. GBM spheroid cultures were cultured as follows, further referred to as CSC medium + growth factors (+ GFs): Advanced DMEM/F12 (Gibco), supplemented with N2 supplement (Invitrogen), 2 mM L-glutamine, 0.15 % D-glucose (Sigma), 100 μM β-mercaptoethanol (Sigma), trace elements B and C (Fisher Scientific), 5 mM HEPES (Life Technologies), 2 μg/ml heparin (Sigma), lipid mixture (Sigma), 10 μg/ml insulin (Sigma), 50 ng/ml human bFGF, and 20 ng/ml human EGF (Peprotech) in ultra-low attachment flasks (Corning). Spheroids were dissociated with accutase (Sigma) and replated in fresh medium twice weekly. In all assays, control medium refers to CSC medium without bFGF and EGF (CSC medium -GFs).

tMVECs were cultured in Endothelial Cell Medium MV2 (Promocell) on gelatine-coated plates (Sigma) and used between passage 2 and 8. HUVECs were cultured in Endothelial Cell Medium MV2 (Promocell).

For preparation of ECCM and HCM, tMVECs or HUVECs were grown to confluence, washed twice with PBS, and CSC medium -GFs was added. After 24 h the conditioned medium was collected, filter-sterilized and stored at −20 °C.

To differentiate GBM CSCs, spheroid cultures were brought to a single cell suspension using accutase and washed twice with CSC medium -GFs. For adherent differentiation as shown in Figs. 2a, 5a, Additional file 1: Figure S3B, Additional file 1: Figure S5, Additional file 1: Figure S6A, Additional file 1: Figure S7, and Additional file 1: Figure S9 GBM cells were plated on laminin-coated coverslips. For all other differentiation experiments, GBM cells were differentiated in ultra-low attachment flasks yielding the same differentiation pattern as adherent differentiation (judged by qRT PCR, see Figs. 2b, 5b, and Additional file 1: Figure S6B); in both conditions for 7 days in CSC medium -GFs containing 100 ng/ml recombinant human BMP4 (+ BMP4; R&D), CSC medium -GFs (GF withdrawal, − GFs), or CSC medium -GFs containing 2 % non-heat inactivated FCS (+2 % FCS). Medium was refreshed after 4 days.

### Clonogenic assays

For clonogenic assays, cells were sorted in low-attachment 96-well plates (Corning) at ascending clonal densities. Fresh medium was added every 3 days and the plates were scored 2 weeks after sorting. Wells containing spheroid structures were scored positive, empty wells or wells containing individual cells were scored negative. Clonogenic potential was determined using the Extreme Limiting Dilution Analysis (ELDA) software: http://bioinf.wehi.edu.au/software/elda/. Data are represented as percent clonogenic potential. Experiments for CD133^+^ cells in control medium and CD133^−^ cells in control medium and ECCM or HCM were carried out simultaneously. Clonogenic assays of differentiated cells were carried out independently and the clonogenic potential of CD133^+^ cells of the respective culture in control medium was included in the graphs as reference but was not determined in the same experiment.

### Immunostaining for FACS, cell sorting, ECCM, HCM and GF treatment, and establishment of cultures upon reversion

GBM spheroid cultures were dissociated into a single cell suspension using accutase and filtering through a 40 μm pore size cell strainer. CD133 (CD133/1 (AC133)-APC (1:25; 130-090-826) or CD133/1 (AC133)-PE (1:100; 130-080-801), Miltenyi Biotec), O4 (O4-APC (1:25), Miltenyi Biotec 130-095-891), and SSEA-1 (1:250, R&D clone MC-480 MAB2155) staining was performed in PBS + 1 % BSA. For SSEA-1 staining, cells were subsequently incubated with an anti-mouse IgM Alexa fluor 488 secondary antibody (1:500, Invitrogen A-21042) in PBS + 1 % BSA. To exclude dead cells, propidium iodide was added to a final concentration of 200 ng/ml. Cells were sorted using the FACS Aria. Gating was performed using an unstained control as reference, exemplary FACS plots are shown in Additional file 1: Figure S1.

For analysis upon ECCM, HCM or GF treatment, cells were sorted into tubes and directly after sorting plated in low-attachment 24-well plates (Corning). Cells were plated in CSC medium containing the GFs at the following concentrations, if not otherwise indicated: 50 ng/ml bFGF, 20 ng/ml EGF, 25 ng/ml human HGF (R&D), and 10 ng/ml human VEGF-121 (Peprotech). After 24 and 72 h or 5 days, spheroids were dissociated with accutase, stained for CD133 and/or O4 and analyzed on the FACS Canto. Gating was performed using the control-treated sample as a reference for the treated samples.

To block bFGF or VEGF in ECCM, a bFGF neutralizing antibody (bFGF nAb 25 μg/ml; clone bFM-1, Millipore 05–117) or Bevacizumab (100 μg/ml; Avastin, Roche), respectively, were added to ECCM and incubated at 37 °C for 1 h before addition of the sorted cells.

To assess the differentiation potential of bFGF-reverted cells, BMP4-differentiated CD133^−^ G062 cells were plated in bFGF-containing medium directly after sorting for 5 days. CD133^−^O4^+^ G073 cells differentiated using GF withdrawal were sorted in clonogenic assays and spheres that formed in the bFGF condition were collected 14 days after sorting. After the 5 day (G062) or 14 day (G073) reversion period in bFGF, spheres were dissociated to a single cell suspension using accutase and grown up in CSC medium + GFs. Once sufficient cells were obtained, the cultures were subjected to adherent differentiation as described above (Additional file 1: Figure S5 for G062 and Additional file 1: Figure S9 for G073).

### Immunofluorescence

GBM cells were plated on laminin-coated coverslips. Cells were fixed using 4 % paraformaldehyde for 20 min on ice. Blocking and primary antibody dilutions were performed in PBS + 0.1 % Triton X-100 + 5 % normal goat serum. Cells were incubated in primary antibody dilutions over night at 4 °C: anti-GFAP (1:250, Sigma G9269), anti-βIII-tubulin (1:100, R&D MAB1195), anti-O4-APC (1:25, Miltenyi Biotec 130-095-891), and anti-Nestin (1:250, Santa Cruz 10C2 sc-23927). Secondary antibody incubation was performed for 1 h at RT (anti-rabbit IgG (H + L) Alexa fluor 488, anti-mouse IgG (H + L) Alexa fluor 488, anti-mouse IgM Alexa fluor 488, each 1:500, Invitrogen A-11034, A-11029, A-21042). For washing steps PBS + 0.1 % Triton X-100 was used. DAPI (Sigma) was included in the last washing step at a concentration of 1 μg/ml. Slides were mounted using ProLong Gold Antifade Reagent (Invitrogen). Pictures were taken at a Zeiss Axiovert 200 M fluorescence microscope at an oil magnification of 63x (scale bars 20 μm).

### Quantitative real-time PCR

RNA was isolated using the Nucleospin RNA II Kit (Macherey-Nagel). For qRT PCR, total RNA was reverse transcribed to cDNA using Superscript III following the manufacturer’s protocol (Invitrogen). qRT PCR was performed using SYBR Green and a Roche Light Cycler 480 II in accordance with the manufacturer’s instructions. All obtained values were normalized to the expression of β-actin; normalization to 18S and B2M yielded similar results. The fold change as compared to GBM cells grown in CSC medium + GFs is shown. Primer sequences: *ACTB*-forward: 5′-CAG AAG GAT TCC TAT GTG GGC GA; *ACTB*-reverse 5′-TTC TCC ATG TCG TCC CAG TTG GT. *GFAP*-forward: 5′- GGC AAA AGC ACC AAA GAC GG; *GFAP*-reverse: 5′-GGC GGC GTT CCA TTT ACA AT. *TUBB3*-forward: 5′-CCT GAC AAT TTC ATC TTT GG TCA GAG T; *TUBB3*-reverse: 5′-GCA CCA CAT CCA GGA CCG AAT. *OLIG1*-forward: 5′-CAC AGC GGC CCG GAG ACT T; *OLIG1*-reverse: 5′-CCT GTA GCC CAC CAG CTC GTA GA. *OLIG2*-forward: 5′-CGC CAG AGC CCG ATG ACC TT; *OLIG2*-reverse: 5′-GAC ACG GTG CCC CCA GTG AA. *Musashi1*-forward: 5′-GAG ACT GAC GCG CCC CAG CC; *Musashi1*-reverse: 5′-CGC CTG GTC CAT GAA AGT GAC G.

### Growth factor array

The growth factor array AAH-GF-1 (RayBiotech) was performed on 3 independently derived ECCM samples and 1 control sample (CSC medium -GFs) following the manufacturer’s instructions. Detection was carried out using the LAS4000 and the spot intensity was quantified using the Odyssey V3.0 program. From each value of the ECCM the value of the control medium was subtracted and the average of the duplicates was calculated. Displayed in the graph are the raw intensities of the 3 different ECCM samples taken together.

### Statistical analyses

Data are presented as means + standard deviation. *P*-values were determined using the unpaired two-tailed Student’s *t*-test in GraphPad Prism 5 software. A *P*-value of <0.05 was considered significant (* *P* < 0.05, ** *P* < 0.01, *** *P* < 0.001).

## Additional file

Additional file 1: Figure S2. ECCM and HCM increase the clonogenic potential of non-stem GBM cells and increase the expression of CD133. (A) Shown is the clonogenic potential of the SSEA-1^−^ fraction of the G073 culture in control medium or ECCM scored 2 weeks after sorting (*n* = 2). (B) Indicated is the clonogenic potential of CD133^−^ G408 cells in control medium or ECCM and of CD133^+^ cells in control medium, scored 2 weeks after sorting (*n* = 2). (C) G408 CD133^−^ cells were plated in control medium or ECCM directly after sorting. Quantification and FACS profiles depict CD133 expression 72 h after sorting (*n* = 2). (D) Displayed is the clonogenic potential of CD133^−^ G602 cells in control medium or HCM and of CD133^+^ cells in control medium, scored 2 weeks after sorting (*n* = 2). (E) G062 CD133^−^ cells were plated in control medium or HCM directly after sorting. Quantification and FACS profiles depict CD133 expression 72 h after sorting (*n* = 4). **Figure S3.** BMP4 differentiation leads to downregulation of SSEA-1 and Nestin and to a decreased clonogenic potential which can be reverted by ECCM and bFGF. (A) Representative FACS plots of SSEA-1 staining on BMP4-differentiated G073 cells and the parental population (+ GFs). The bar plot shows % SSEA-1^+^ cells upon BMP4-induced differentiation (+ BMP4) compared to the parental population (+ GFs) (*n* =5). (B) 7 day BMP4 differentiation leads to the downregulation of Nestin expression in G073 (left) and G062 (right) cells as compared to cells plated in CSC medium + GFs (scale bars 20 μm; *n* = 2). (C) The clonogenic potential of BMP4-differentiated G073 (left) and G062 (right) cells upon plating in control medium, ECCM or medium containing bFGF was determined using clonogenic assays (*n* = 2). (D) GFAP and βIII-tubulin expression in 7 day BMP4-differentiated G062 cells and in spheres formed in the bFGF condition shown in (C). Depicted is the fold change compared to cells plated in CSC medium + GFs. 1 representative of 3 independent experiments is shown. **Figure S4.** bFGF is secreted by tMVECs and induces CD133 expression on BMP4-differentiated CD133^−^ cells. (A) Quantification of the growth factor arrays shown in Fig. 3a. Graph depicts quantification of 3 growth factor arrays using different batches of ECCM. Shown is the intensity of each spot minus the intensity of the respective spot on the control array. (B) BMP4-differentiated CD133^−^ G062 cells were plated in the indicated condition directly after sorting. 5 days after sorting CD133 expression was analyzed by FACS (*n* = 4). **Figure S5.** Neurospheres derived from CD133^−^ G062 cells stimulated with bFGF recapitulate the differentiation pattern of the parental population. (A) Plating G062 cells for 7 days in BMP4 containing medium leads to morphological differentiation as well as upregulation of GFAP, indicating the differentiation to the astrocytic lineage as compared to cells plated in GF-containing medium (scale bars 20 μm). (B) A culture derived from BMP4-differentiated CD133^−^ G062 cells stimulated with bFGF after sorting was plated in medium containing GFs or BMP4. These cells displayed the same differentiation pattern as the parental population, demonstrated by the expression of GFAP and βIII-tubulin (*n* = 2, scale bars 20 μm). **Figure S6.** Differentiated GBM oligodendrocytes can be phenotypically and functionally redirected to the CSC state by bFGF. (A) Differentiation for 7 days by addition of 2 % FCS (+2 % FCS). G073 cells differentiate into all 3 lineages, indicated by the expression of GFAP, βIII-tubulin, and O4 (scale bars 20 μm). (B) Analysis of differentiation and CSC markers by qRT PCR. Depicted is the fold change compared to cells plated in CSC medium + GFs. 1 representative of 3 independent experiments is shown. (C) Differentiated GBM cells express the oligodendrocyte marker O4 on their surface as determined by FACS. (D) CD133^−^O4^+^ cells were sorted and plated in the indicated condition. 5 days after sorting CD133 and O4 expression were analyzed by FACS (*n* = 3). (E) The clonogenic potential of differentiated CD133^−^O4^+^ cells was determined using clonogenic assays (*n* = 4). (F) CD133 and O4 expression of spheres formed in (E) in the bFGF condition compared to the non-differentiated parental GBM spheroid culture (*n* = 2). **Figure S7.** Nestin expression is downregulated upon differentiation in G073 cells. 7 day differentiation of G073 cells using GF withdrawal (−GF) or addition of 2 % FCS (+2 % FCS) leads to downregulation of Nestin expression as compared to cells plated in CSC medium + GFs (scale bars 20 μm; *n* = 2). **Figure S8.** Gain of CD133 and loss of O4 expression on differentiated cells induced by bFGF is not due to expansion of a contaminating CD133^+^O4^−^ fraction. (A) Differentiation and sorting of G073 cells as described in (Fig. 5 and Additional file 1: Figure S6). The purity of the sort was analyzed immediately afterwards by FACS. (B) After differentiation using GF withdrawal, CD133^−^O4^+^ cells were sorted twice and plated in low adherence for 5 days. Subsequently the expression of CD133 and O4 was analyzed by FACS. Induction of CD133 and loss of O4 were as efficient as after a single sort (*n* = 1). **Figure S9.** Neurospheres derived from CD133^−^O4^+^ cells stimulated with bFGF recapitulate the differentiation pattern of the parental population*.* Cultures derived from G073 spheres that formed in a clonogenic assay in bFGF-containing medium (as described in Fig. 5e) were differentiated using the depicted conditions. These cells displayed the same differentiation pattern as the parental population, demonstrated by the expression of GFAP, βIII-tubulin, and O4 (*n* = 3; scale bars 20 μm). **Figure S10.** Differentiated GBM oligodendrocytes can be phenotypically and functionally redirected to the CSC state by ECCM. (A,B) Differentiation of G073 cells for 7 days by GF withdrawal (− GFs) (A) or addition of 2 % FCS (B) and subsequent sorting of CD133^−^O4^+^ cells in the indicated condition. 5 days after sorting CD133 and O4 expression were reanalyzed by FACS (*n* = 4 for - GF and *n* = 2 for + 2 % FCS) (C,D) The clonogenic potential of differentiated CD133^−^O4^+^ cells was determined using clonogenic assays (*n* = 2 for - GF and *n* = 3 for + 2 % FCS). **Figure S1.** Gating on cell populations for sorting was performed using an unstained control as reference. (A-C) Shown are exemplary FACS plots for the identification of (A) the CD133^−^O4^+^ (− GF and + 2 % FCS) and CD133^−^ (+ BMP4) fraction of G073 cells, (B) the SSEA-1^−^ population of G073 cells, and (C) the CD133^−^ fraction of BMP4-differentiated G062 cells.

## Abbreviations

GBM: Glioblastoma multiforme
CSCs: Cancer stem cells
tMVECs: Tumor microvascular endothelial cells
bFGF: Basic fibroblast growth factor
TMZ: Temozolomide
ECCM: Endothelial cell conditioned medium
SSEA-1: Stage-specific embryonic antigen 1
HUVECs: Human umbilical vein endothelial cells
HCM: HUVEC conditioned medium
BMP4: Bone morphogenetic protein 4
GFAP: Glial fibrillary acidic protein
qRT PCR: Quantitative real-time PCR
TUBB3: Tubulin, beta 3 class III
OLIG: Oligodendrocyte transcription factor
GFs: Growth factors
EGF: Epidermal growth factor
HGF: Hepatocyte growth factor
VEGF: Vascular endothelial growth factor
bFGF nAb: bFGF neutralizing antibody
IGFBPs: Insulin-like growth factor-binding proteins
FCS: Fetal calf serum
FACS: Fluorescence activated cell sorting

## References

[1] Stupp R, Hegi ME, Mason WP, van den Bent MJ, Taphoorn MJ, Janzer RC (2009). Effects of radiotherapy with concomitant and adjuvant temozolomide versus radiotherapy alone on survival in glioblastoma in a randomised phase III study: 5-year analysis of the EORTC-NCIC trial. Lancet Oncol.

[2] Clarke MF, Dick JE, Dirks PB, Eaves CJ, Jamieson CHM, Jones DL (2006). Cancer stem cells--perspectives on current status and future directions: AACR Workshop on cancer stem cells. Cancer Res.

[3] Bao S, Wu Q, McLendon RE, Hao Y, Shi Q, Hjelmeland AB (2006). Glioma stem cells promote radioresistance by preferential activation of the DNA damage response. Nature.

[4] Colak S, Zimberlin C, Fessler E, Hogdal L, Prasetyanti P, Grandela C (2014). Decreased mitochondrial priming determines chemoresistance of colon cancer stem cells. Cell Death Differ.

[5] Vermeulen L, De Sousa E Melo F, van der Heijden M, Cameron K, de Jong JH, Borovski T (2010). Wnt activity defines colon cancer stem cells and is regulated by the microenvironment. Nat Cell Biol.

[6] Vermeulen L, De Sousa E Melo F, Richel DJ, Medema JP (2012). The developing cancer stem-cell model: clinical challenges and opportunities. Lancet Oncol.

[7] Fessler E, Dijkgraaf FE, De Sousa E Melo F, Medema JP (2013). Cancer stem cell dynamics in tumor progression and metastasis: is the microenvironment to blame?. Cancer Lett.

[8] Gupta PB, Fillmore CM, Jiang G, Shapira SD, Tao K, Kuperwasser C, et al. Stochastic state transitions give rise to phenotypic equilibrium in populations of cancer cells. Cell. 2011;146:633–44. 10.1016/j.cell.2011.07.026 21854987

[9] Roesch A, Fukunaga-Kalabis M, Schmidt EC, Zabierowski SE, Brafford PA, Vultur A (2010). A temporarily distinct subpopulation of slow-cycling melanoma cells is required for continuous tumor growth. Cell.

[10] Li Y, Laterra J (2012). Cancer stem cells: distinct entities or dynamically regulated phenotypes?. Cancer Res.

[11] Heddleston JM, Li Z, McLendon RE, Hjelmeland AB, Rich JN (2009). The hypoxic microenvironment maintains glioblastoma stem cells and promotes reprogramming towards a cancer stem cell phenotype. Cell Cycle.

[12] Hjelmeland AB, Wu Q, Heddleston J, Choudhary G, MacSwords J, Lathia J (2011). Acidic stress promotes a glioma stem cell phenotype. Cell Death Differ.

[13] Auffinger B, Tobias AL, Han Y, Lee G, Guo D, Dey M (2014). Conversion of differentiated cancer cells into cancer stem-like cells in a glioblastoma model after primary chemotherapy. Cell Death Differ.

[14] Dahan P, Martinez Gala J, Delmas C, Monferran S, Malric L, Zentkowski D (2014). Ionizing radiations sustain glioblastoma cell dedifferentiation to a stem-like phenotype through survivin: possible involvement in radioresistance. Cell Death Dis.

[15] Chaffer CL, Marjanovic N, Lee T, Bell G, Kleer C, Reinhard F (2013). Poised chromatin at the ZEB1 promoter enables breast cancer cell plasticity and enhances tumorigenicity. Cell.

[16] Chaffer CL, Brueckmann I, Scheel C, Kaestli AJ, Wiggins PA, Rodrigues LO (2011). Normal and neoplastic nonstem cells can spontaneously convert to a stem-like state. Proc Natl Acad Sci U S A.

[17] Singh SK, Clarke ID, Terasaki M, Bonn VE, Hawkins C, Squire J (2003). Identification of a cancer stem cell in human brain tumors. Cancer Res.

[18] Singh SK, Hawkins C, Clarke ID, Squire JA, Bayani J, Hide T (2004). Identification of human brain tumour initiating cells. Nature.

[19] Beier D, Hau P, Proescholdt M, Lohmeier A, Wischhusen J, Oefner P (2007). CD133(+) and CD133(−) glioblastoma-derived cancer stem cells show differential growth characteristics and molecular profiles. Cancer Res.

[20] Wang J, Sakariassen P, Tsinkalovsky O, Immervoll H, Bøe S, Svendsen A (2008). CD133 negative glioma cells form tumors in nude rats and give rise to CD133 positive cells. Int J Cancer.

[21] Chen R, Nishimura MC, Bumbaca SM, Kharbanda S, Forrest WF, Kasman IM (2010). A hierarchy of self-renewing tumor-initiating cell types in glioblastoma. Cancer Cell.

[22] Ogden A, Waziri A, Lochhead R, Fusco D, Lopez K, Ellis J (2008). Identification of A2B5 + CD133- tumor-initiating cells in adult human gliomas. Neurosurgery.

[23] Borovski T, Beke P, van Tellingen O, Rodermond HM, Verhoeff J, Lascano V (2012). Therapy-resistant tumor microvascular endothelial cells contribute to treatment failure in glioblastoma multiforme. Oncogene.

[24] Son M, Woolard K, Nam D, Lee J, Fine H (2009). SSEA-1 is an enrichment marker for tumor-initiating cells in human glioblastoma. Cell Stem Cell.

[25] Piccirillo SGM, Reynolds B, Zanetti N, Lamorte G, Binda E, Broggi G (2006). Bone morphogenetic proteins inhibit the tumorigenic potential of human brain tumour-initiating cells. Nature.

[26] Lee J, Kotliarova S, Kotliarov Y, Li A, Su Q, Donin NM (2006). Tumor stem cells derived from glioblastomas cultured in bFGF and EGF more closely mirror the phenotype and genotype of primary tumors than do serum-cultured cell lines. Cancer Cell.

[27] Haley EM, Kim Y (2014). The role of basic fibroblast growth factor in glioblastoma multiforme and glioblastoma stem cells and in their in vitro culture. Cancer Lett.

[28] Hamerlik P, Lathia JD, Rasmussen R, Wu Q, Bartkova J, Lee M (2012). Autocrine VEGF-VEGFR2-Neuropilin-1 signaling promotes glioma stem-like cell viability and tumor growth. J Exp Med.

[29] Hsieh D, Hsieh A, Stea B, Ellsworth R (2010). IGFBP2 promotes glioma tumor stem cell expansion and survival. Biochem Biophys Res Commun.

[30] Lathia J, Gallagher J, Heddleston J, Wang J, Eyler C, Macswords J (2010). Integrin alpha 6 regulates glioblastoma stem cells. Cell Stem Cell.

[31] Borovski T, Verhoeff JJC, ten Cate R, Cameron K, de Vries N, van Tellingen O (2009). Tumor microvasculature supports proliferation and expansion of glioma-propagating cells. Int J Cancer.

[32] Verhaak RG, Hoadley KA, Purdom E, Wang V, Qi Y, Wilkerson MD (2010). Integrated genomic analysis identifies clinically relevant subtypes of glioblastoma characterized by abnormalities in PDGFRA, IDH1, EGFR, and NF1. Cancer Cell.

[33] Phillips HS, Kharbanda S, Chen R, Forrest WF, Soriano RH, Wu TD (2006). Molecular subclasses of high-grade glioma predict prognosis, delineate a pattern of disease progression, and resemble stages in neurogenesis. Cancer Cell.

