# Supplementary material for: Endothelial cells induce cancer stem cell features in differentiated glioblastoma cells via bFGF
Source: Mol Cancer. 2015 Aug 19;14:157. doi: 10.1186/s12943-015-0420-3 (PMC4539660; doi:10.1186/s12943-015-0420-3)
Supplement: Additional file 1: Figure S2. — ECCM and HCM increase the clonogenic potential of non-stem GBM cells and increase the expression of CD133. (A) Shown is the clonogenic potential of the SSEA-1− fraction of the G073 culture in control medium or ECCM scored 2 weeks after sorting (n = 2). (B) Indicated is the clonogenic potential of CD133− G408 cells in control medium or ECCM and of CD133+ cells in control medium, scored 2 weeks after sorting (n = 2). (C) G408 CD133− cells were plated in control medium or ECCM directly after sorting. Quantification and FACS profiles depict CD133 expression 72 h after sorting (n = 2). (D) Displayed is the clonogenic potential of CD133− G602 cells in control medium or HCM and of CD133+ cells in control medium, scored 2 weeks after sorting (n = 2). (E) G062 CD133− cells were plated in control medium or HCM directly after sorting. Quantification and FACS profiles depict CD133 expression 72 h after sorting (n = 4). Figure S3. BMP4 differentiation leads to downregulation of SSEA-1 and Nestin and to a decreased clonogenic potential which can be reverted by ECCM and bFGF. (A) Representative FACS plots of SSEA-1 staining on BMP4-differentiated G073 cells and the parental population (+ GFs). The bar plot shows % SSEA-1+ cells upon BMP4-induced differentiation (+ BMP4) compared to the parental population (+ GFs) (n =5). (B) 7 day BMP4 differentiation leads to the downregulation of Nestin expression in G073 (left) and G062 (right) cells as compared to cells plated in CSC medium + GFs (scale bars 20 μm; n = 2). (C) The clonogenic potential of BMP4-differentiated G073 (left) and G062 (right) cells upon plating in control medium, ECCM or medium containing bFGF was determined using clonogenic assays (n = 2). (D) GFAP and βIII-tubulin expression in 7 day BMP4-differentiated G062 cells and in spheres formed in the bFGF condition shown in (C). Depicted is the fold change compared to cells plated in CSC medium + GFs. 1 representative of 3 independent experiments is shown. Figure S4. bFGF [file 12943_2015_420_MOESM1_ESM.pdf]

Figure S2

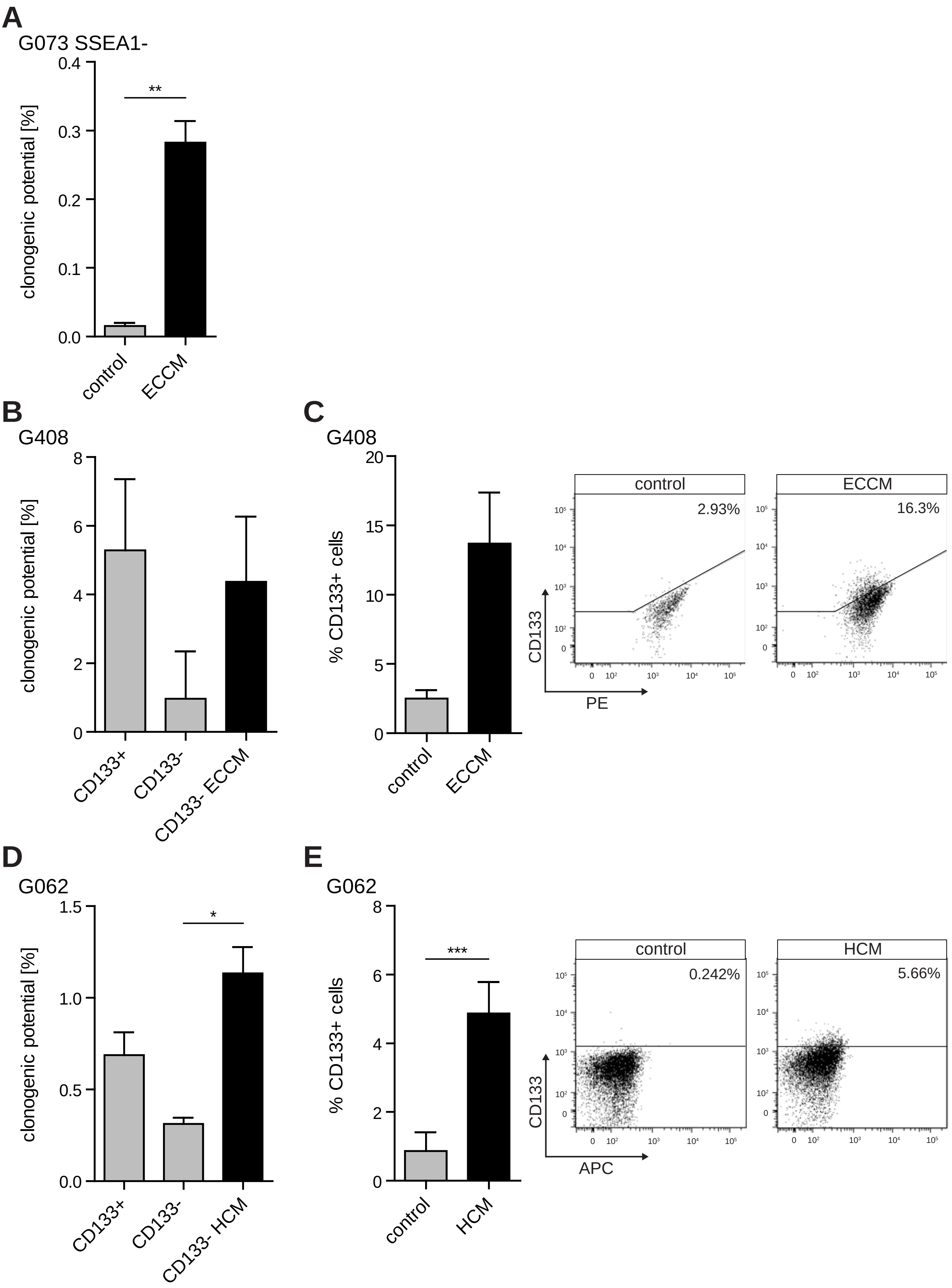

A
G073 SSEA1-
clonogenic potential [%]
0.0
0.1
0.2
0.3
0.4
**
control
ECCM
B
G408
clonogenic potential [%]
0
2
4
6
8
CD133+
CD133-
CD133- ECCM
C
G408
% CD133+ cells
0
5
10
15
20
control
ECCM
control
2.93%
ECCM
16.3%
CD133
PE
D
G062
clonogenic potential [%]
0.0
0.5
1.0
1.5
*
CD133+
CD133-
CD133- HCM
E
G062
% CD133+ cells
0
2
4
6
8
***
control
HCM
control
0.242%
HCM
5.66%
CD133
APC

Figure S3

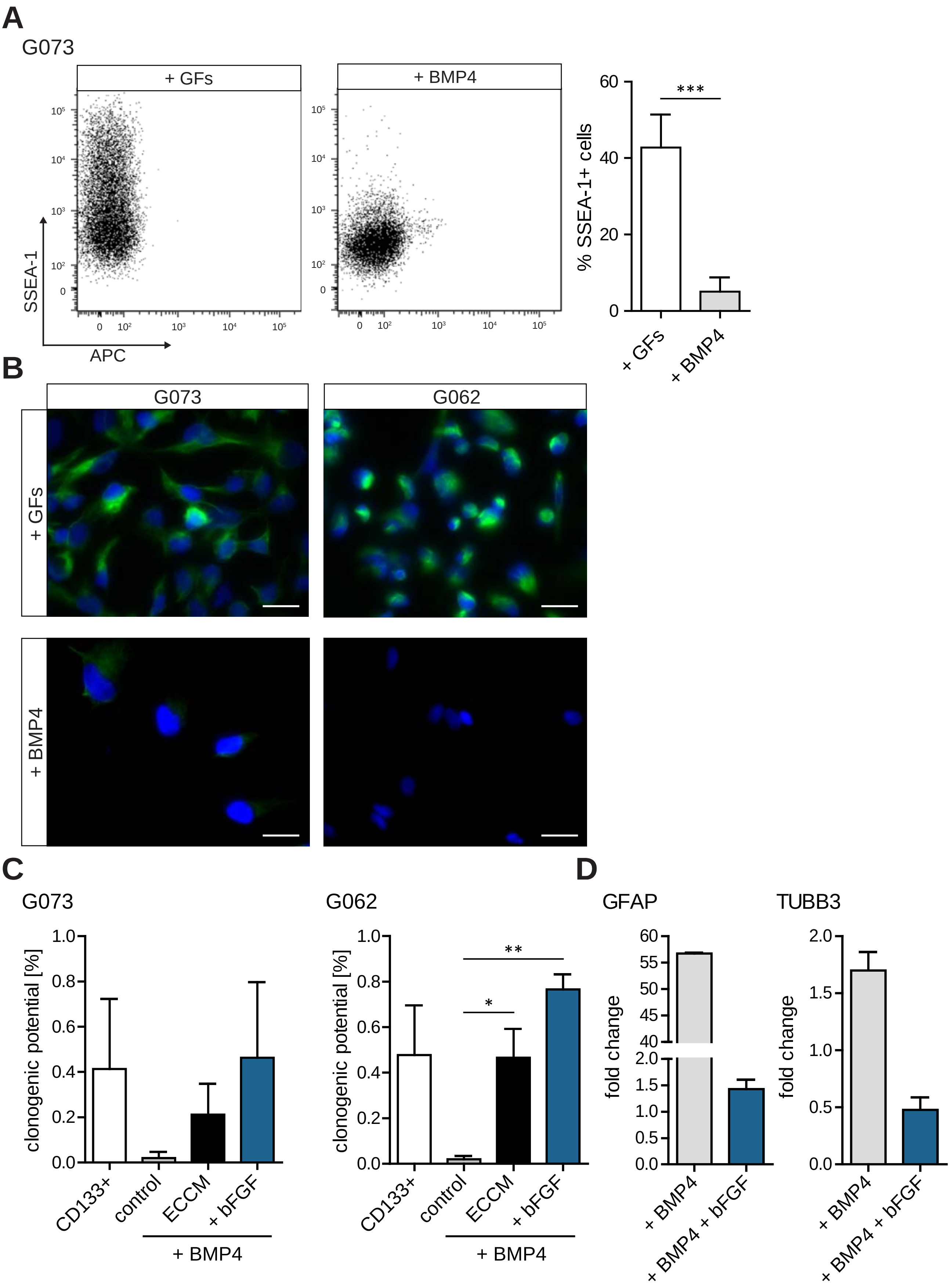

A
G073
+ GFs
+ BMP4
SSEA-1
APC
% SSEA-1+ cells
***
+ GFs
+ BMP4
B
G073
G062
+ GFs
+ BMP4
C
G073
G062
clonogenic potential [%]
CD133+
control
ECCM
+ bFGF
+ BMP4
*
**
D
GFAP
TUBB3
fold change
+ BMP4
+ BMP4 + bFGF

Figure S4

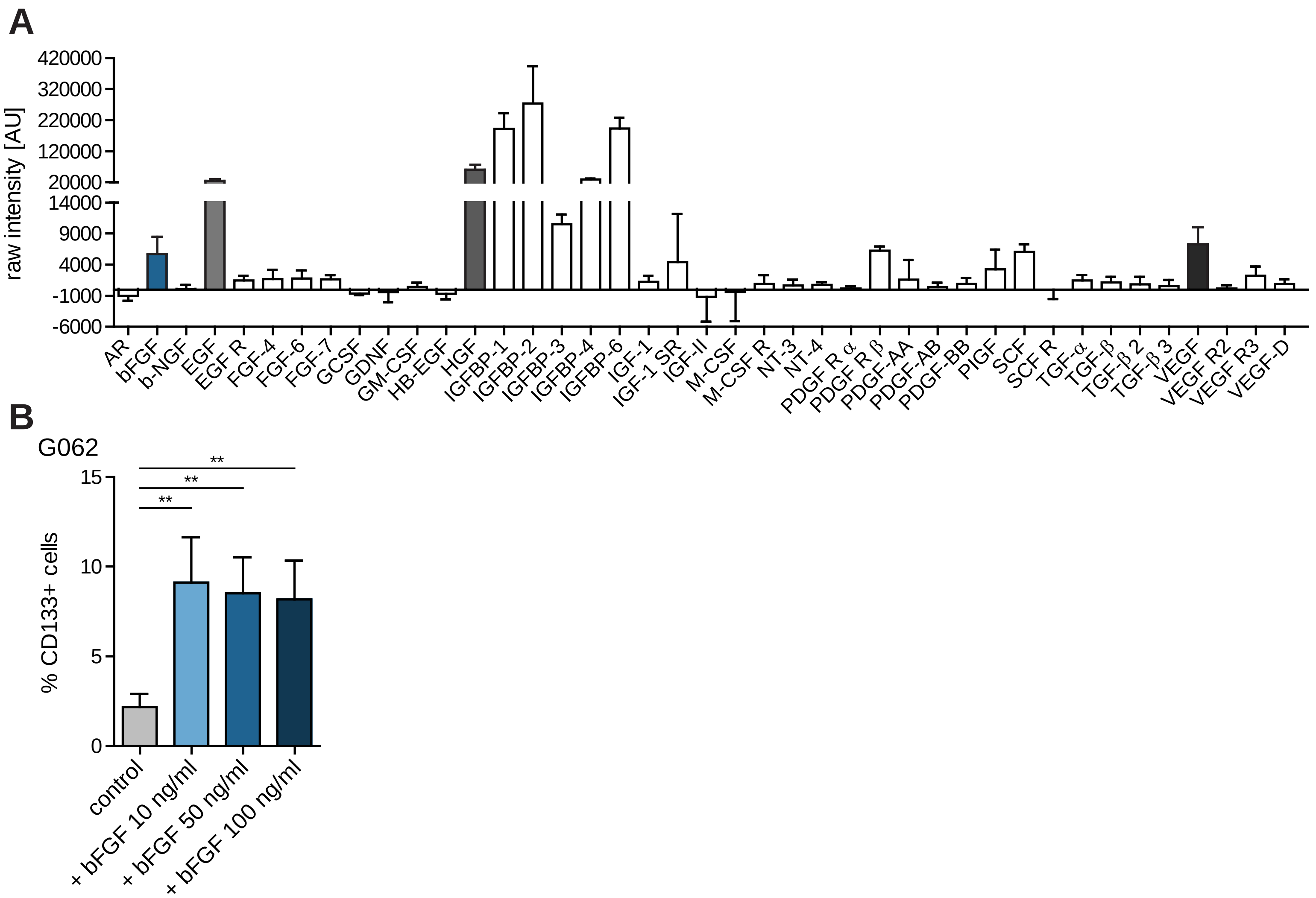
A
raw intensity [AU]
420000
320000
220000
120000
20000
14000
9000
4000
-1000
-6000
AR
bFGF
b-NGF
EGF
EGF R
FGF-4
FGF-6
FGF-7
GCSF
GDNF
GM-CSF
HB-EGF
HGF
IGFBP-1
IGFBP-2
IGFBP-3
IGFBP-4
IGFBP-6
IGF-1
IGF-1 SR
IGF-II
M-CSF
M-CSF R
NT-3
NT-4
PDGF R α
PDGF R β
PDGF-AA
PDGF-AB
PDGF-BB
PIGF
SCF
SCF R
TGF-α
TGF-β
TGF-β 2
TGF-β 3
VEGF
VEGF R2
VEGF R3
VEGF-D
B
G062
% CD133+ cells
15
10
5
0
**
**
**
control
+ bFGF 10 ng/ml
+ bFGF 50 ng/ml
+ bFGF 100 ng/ml

Figure S5

A

parental

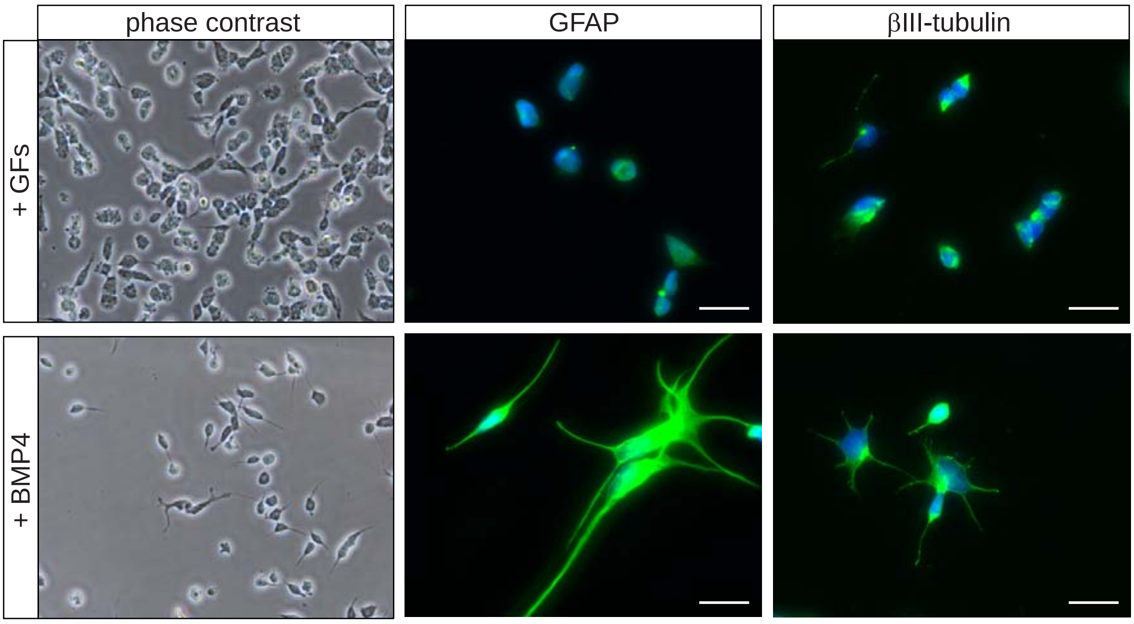

B

bFGF-reverted

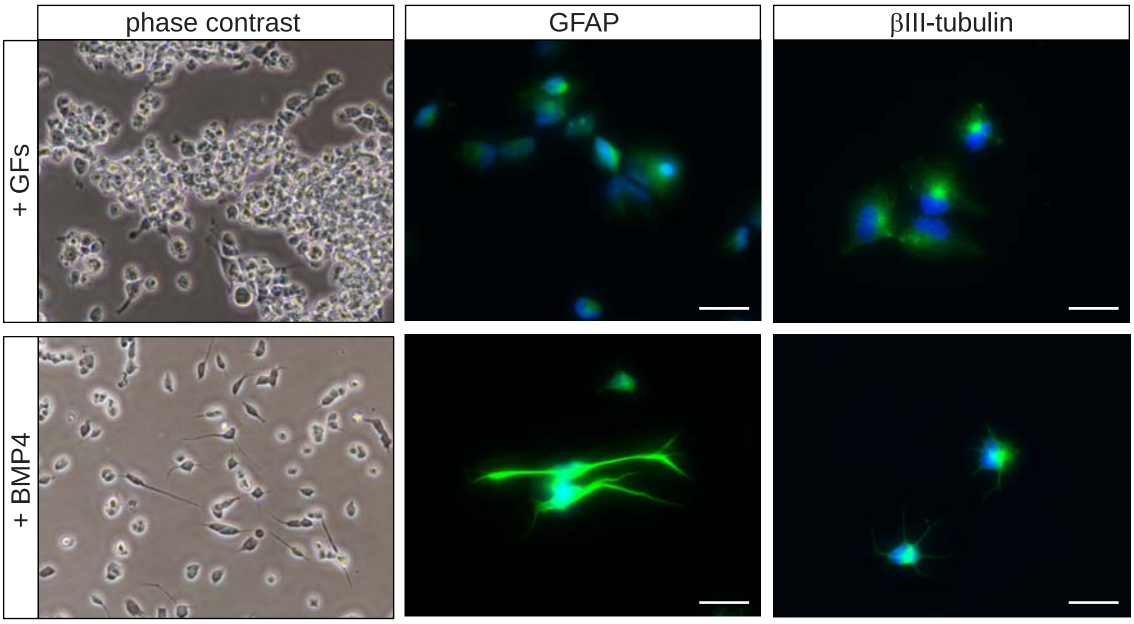

Figure S6

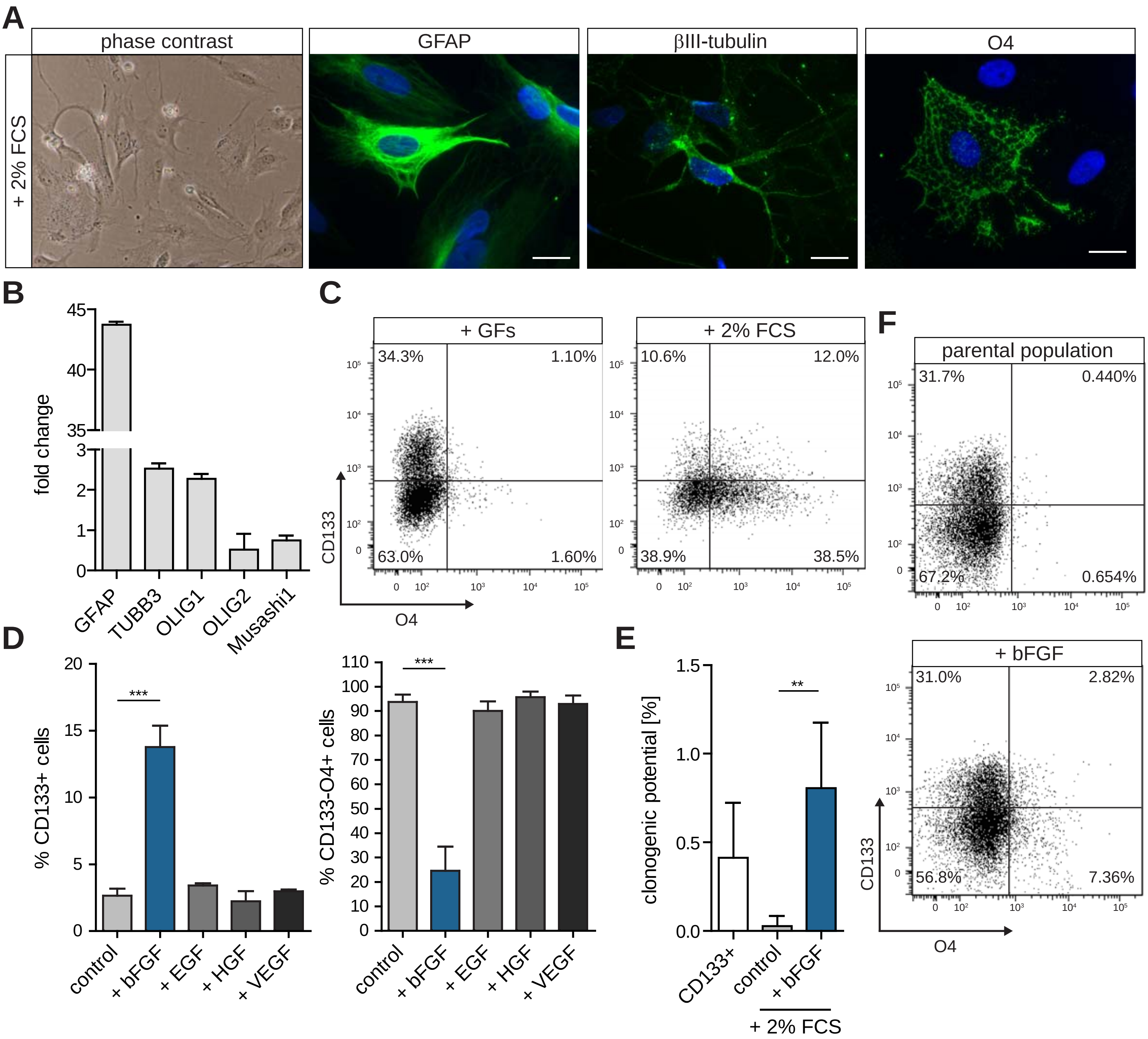

A
phase contrast
GFAP
βIII-tubulin
O4
+ 2% FCS
B
fold change
45
40
35
3
2
1
0
GFAP
TUBB3
OLIG1
OLIG2
Musashi1
C
+ GFs
34.3%
1.10%
63.0%
1.60%
+ 2% FCS
10.6%
12.0%
38.9%
38.5%
CD133
O4
F
parental population
31.7%
0.440%
67.2%
0.654%
+ bFGF
31.0%
2.82%
56.8%
7.36%
CD133
O4
D
% CD133+ cells
***
control
+ bFGF
+ EGF
+ HGF
+ VEGF
% CD133-O4+ cells
***
control
+ bFGF
+ EGF
+ HGF
+ VEGF
E
clonogenic potential [%]
**
CD133+
control
+ bFGF
+ 2% FCS

Figure S7

G073

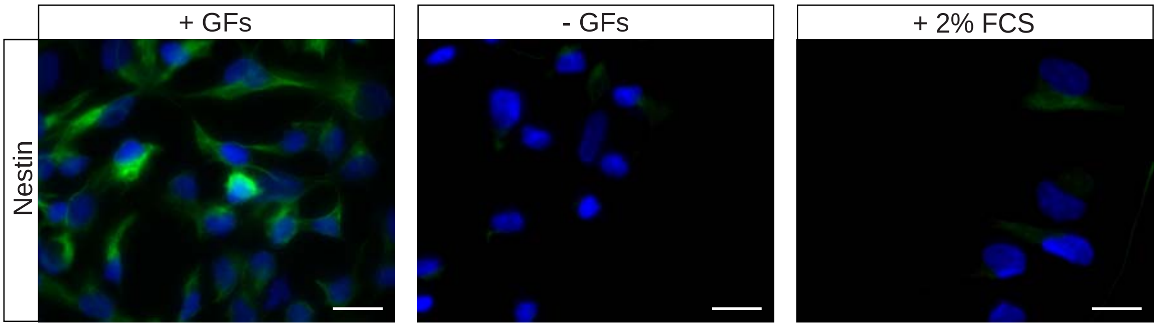

Figure S8

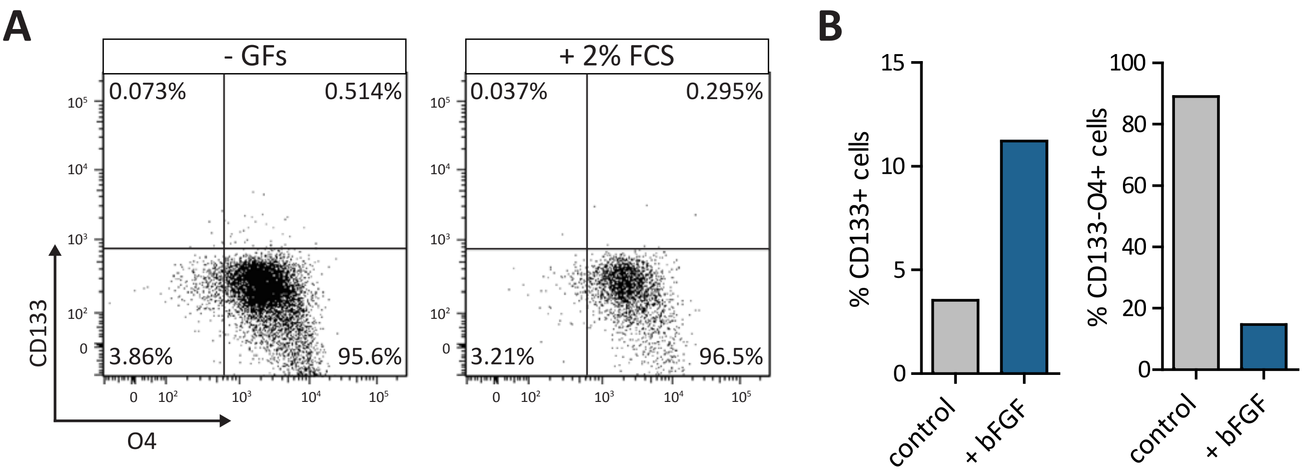
A
- GFs
+ 2% FCS
0.073%
0.514%
3.86%
95.6%
0.037%
0.295%
3.21%
96.5%
CD133
O4
B
% CD133+ cells
% CD133-O4+ cells
control
+ bFGF
15
10
5
0
100
80
60
40
20
0

Figure S9

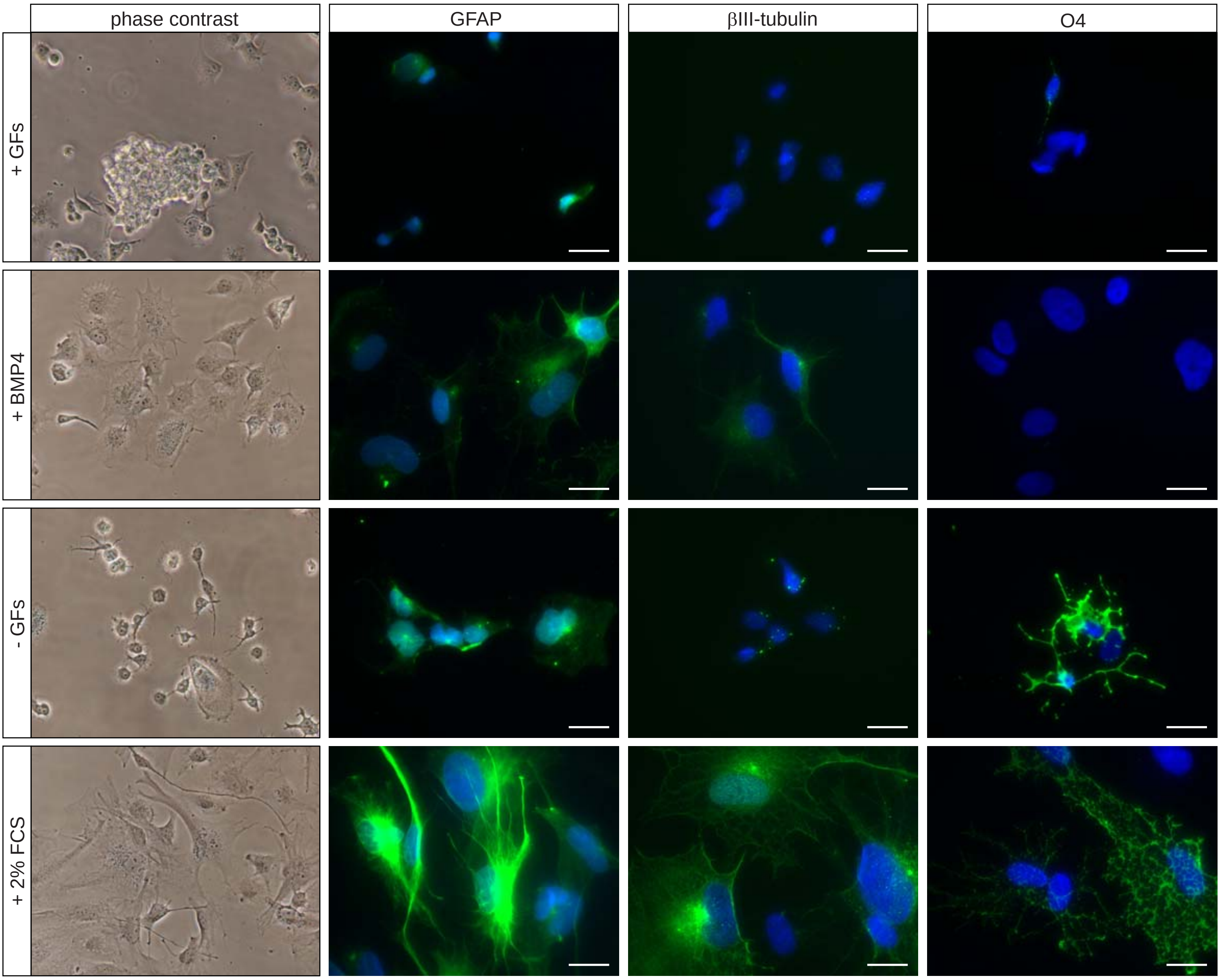

Figure S10

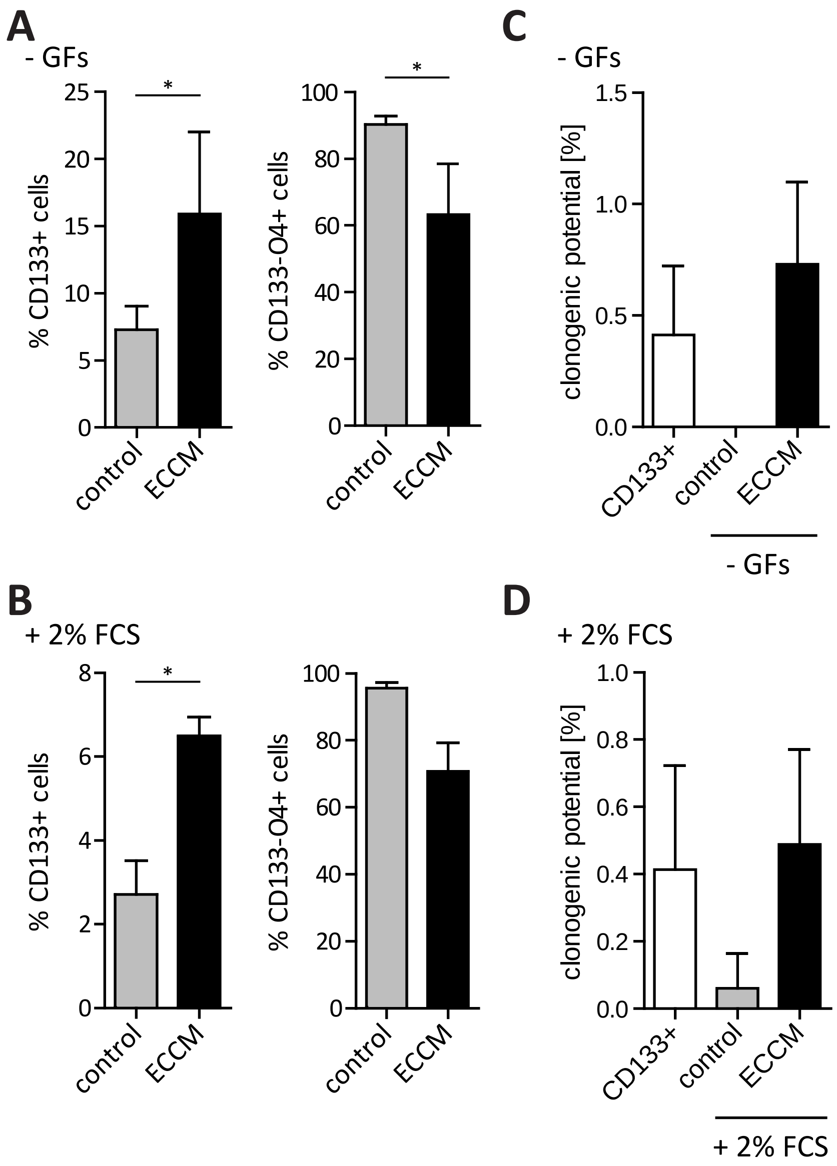
A
- GFs
*
% CD133+ cells
25
20
15
10
5
0
control
ECCM
% CD133-O4+ cells
100
80
60
40
20
0
control
ECCM
C
- GFs
clonogenic potential [%]
1.5
1.0
0.5
0.0
CD133+
control
ECCM
- GFs
B
+ 2% FCS
*
% CD133+ cells
8
6
4
2
0
control
ECCM
% CD133-O4+ cells
100
80
60
40
20
0
control
ECCM
D
+ 2% FCS
clonogenic potential [%]
1.0
0.8
0.6
0.4
0.2
0.0
CD133+
control
ECCM
+ 2% FCS

Figure S1

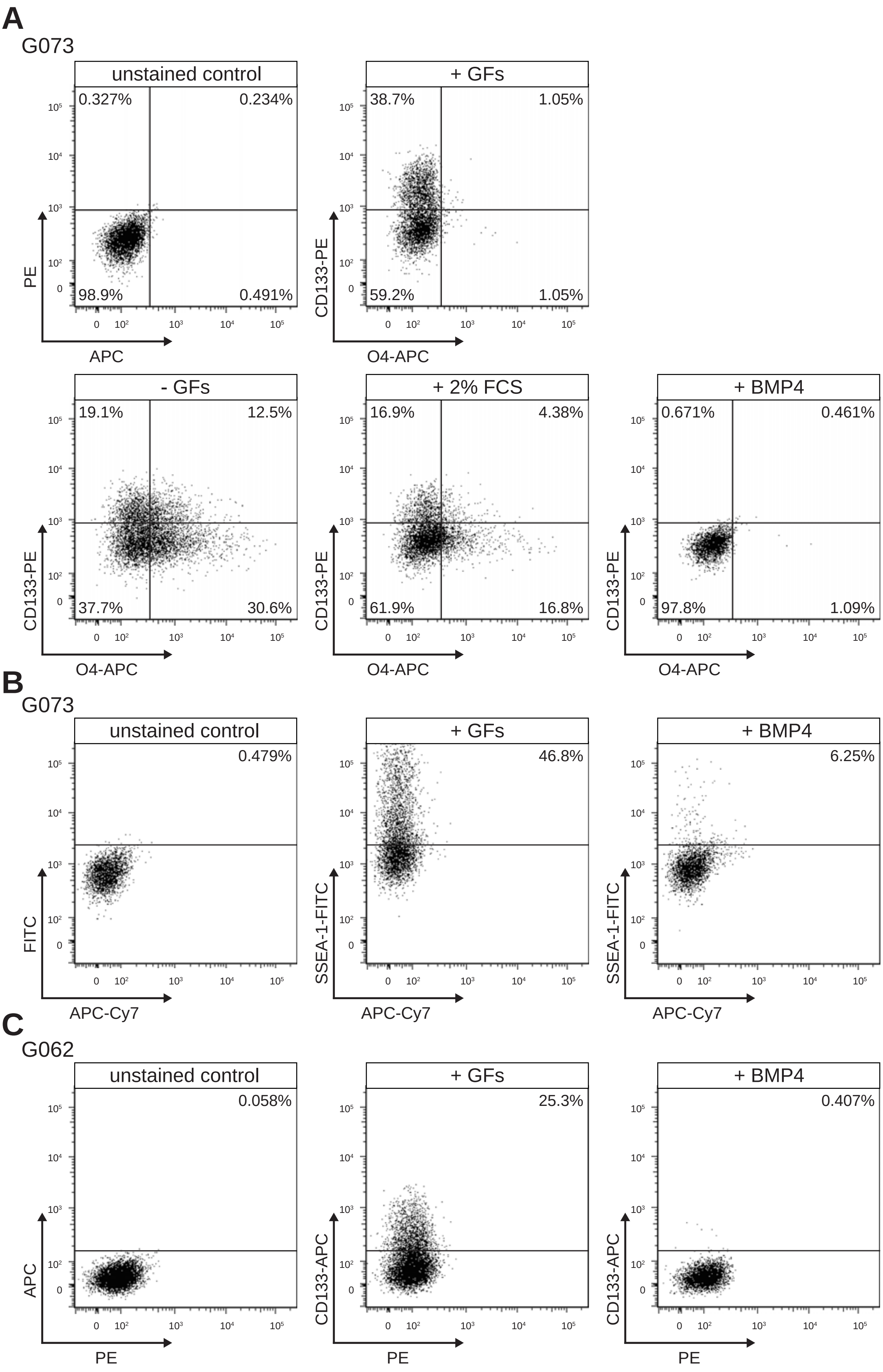
A
G073
unstained control
0.327%
0.234%
98.9%
0.491%
PE
APC
+ GFs
38.7%
1.05%
59.2%
1.05%
CD133-PE
O4-APC
- GFs
19.1%
12.5%
37.7%
30.6%
+ 2% FCS
16.9%
4.38%
61.9%
16.8%
+ BMP4
0.671%
0.461%
97.8%
1.09%
B
G073
unstained control
0.479%
FITC
APC-Cy7
+ GFs
46.8%
SSEA-1-FITC
+ BMP4
6.25%
C
G062
unstained control
0.058%
APC
PE
+ GFs
25.3%
CD133-APC
+ BMP4
0.407%
